# Increase in DNA Damage by MYCN Knockdown Through Regulating Nucleosome Organization and Chromatin State in Neuroblastoma

**DOI:** 10.3389/fgene.2019.00684

**Published:** 2019-07-24

**Authors:** Xinjie Hu, Weisheng Zheng, Qianshu Zhu, Liang Gu, Yanhua Du, Zhe Han, Xiaobai Zhang, Daniel R. Carter, Belamy B. Cheung, Andong Qiu, Cizhong Jiang

**Affiliations:** ^1^Institute of Translational Research, Tongji Hospital, the School of Life Sciences and Technology, Shanghai Key Laboratory of Signaling and Disease Research, Tongji University, Shanghai, China; ^2^The Research Center of Stem Cells and Ageing, Tsingtao Advanced Research Institute, Tongji University, Tsingdao, China; ^3^Children’s Cancer Institute Australia, Lowy Cancer Research Centre, UNSW Sydney, Kensington, NSW, Australia; ^4^School of Women’s and Children’s Health, UNSW Sydney, Randwick, NSW, Australia; ^5^School of Biomedical Engineering, University of Technology, Sydney, NSW, Australia

**Keywords:** neuroblastoma, MYCN, nucleosome, histone modification, DNA repair

## Abstract

As a transcription factor, MYCN regulates myriad target genes including the histone chaperone FACT. Moreover, FACT and MYCN expression form a forward feedback loop in neuroblastoma. It is unclear whether MYCN is involved in chromatin remodeling in neuroblastoma through regulation of its target genes. We showed here that MYCN knockdown resulted in loss of the nucleosome-free regions through nucleosome assembly in the promoters of genes functionally enriched for DNA repair. The active mark H3K9ac was removed or replaced by the repressive mark H3K27me3 in the promoters of double-strand break repair-related genes upon MYCN knockdown. Such chromatin state alterations occurred only in MYCN-bound promoters. Consistently, MYCN knockdown resulted in a marked increase in DNA damage in the treatment with hydroxyurea. In contrast, nucleosome reorganization and histone modification changes in the enhancers largely included target genes with tumorigenesis-related functions such as cell proliferation, cell migration, and cell–cell adhesion. The chromatin state significantly changed in both MYCN-bound and MYCN-unbound enhancers upon MYCN knockdown. Furthermore, MYCN knockdown independently regulated chromatin remodeling in the promoters and the enhancers. These findings reveal the novel epigenetic regulatory role of MYCN in chromatin remodeling and provide an alternative potential epigenetic strategy for MYCN-driven neuroblastoma treatment.

## Introduction

Neuroblastoma is a cancer that arises in neural-crest tissues, typically in sympathetic ganglia and adrenal glands. It generally occurs in children under 5 years of age, with the median age at diagnosis of about 17 months (London et al., 2005), with an incidence of dozens of cases per million children (Stiller and Parkin, 1992; Maris, 2010). Neuroblastoma is the most malignant and common solid tumor diagnosed in the first year of life (Maris, 2010). It accounts for the disproportionately high mortality among the cancers of childhood.

The *c-MYC* homolog *MYCN* (encoding the transcription factor N-MYC), a proto-oncogene, was originally isolated from neuroblastoma cells (Kohl et al., 1983; Schwab et al., 1983). MYCN contains a basic helix-loop-helix (bHLH) domain. It binds to DNA as a dimer with another bHLH protein. MYCN plays critical roles in regulating many cellular processes such as cell growth, differentiation, and apoptosis. *MYCN* amplification is often associated with a variety of tumors, mostly neuroblastoma. The overexpression of *MYCN* contributes to the genesis of neuroblastoma in the transgenic mice (Weiss et al., 1997). It was reported that *MYCN* expression was required to activate the differentiation in neuroblastoma cells (Guglielmi et al., 2014). Approximately 25% of neuroblastoma tumors harbor amplification of *MYCN* (Matthay et al., 2016) that strongly correlates with a poor prognosis (Janoueix-Lerosey et al., 2009). *MYCN* amplification is also often associated with genetic variations such as segmental chromosomal loss (Thompson et al., 2016). Consistently, the most malignant neuroblastoma contains amplification of *MYCN* (Matthay et al., 2016). Therefore, *MYCN* amplification is a significant predictor of poor clinical outcome in neuroblastoma patients.

As a transcription factor, MYCN regulates transcription of many genes. Based on the MYC target gene signature, we identified FACT (facilitates chromatin transcription), encoding a histone chaperone, as a therapeutic target in neuroblastoma (Carter et al., 2015). Human FACT consists of two subunits Spt16 and SSRP1 (Orphanides et al., 1999). FACT is highly conserved in eukaryotes (Brewster et al., 1998). Notably, FACT as a histone chaperone plays a critical role in chromatin architecture through regulating nucleosome assembly and eviction, for example, stability of H2A-H2B dimer (Orphanides et al., 1998; Reinberg and Sims, 2006; Winkler and Luger, 2011; Formosa, 2012). FACT also functioned in DAN repair by activating p53 and linking to H2AX (Heo et al., 2008). Interestingly, MYCN and FACT expression formed a positive feedback loop in neuroblastoma cells. Inhibition of MYCN down-regulated FACT expression in neuroblastoma cells, and *vice versa* (Carter et al., 2015). Thus, it is very likely that MYCN can also alter chromatin state indirectly through its target genes. However, this remains unexplored in neuroblastoma.

To address these questions, we knocked down MYCN in the human neuroblastoma cell line BE(2)C and profiled the transcriptome, the genome-wide nucleosome occupancy, and key histone modification signals. Epigenomics analyses revealed that MYCN knockdown altered nucleosome reorganization and histone modifications. The chromatin remodeling induced by MYCN knockdown was functionally enriched for cell proliferation and DNA repair, creating a synthetic lethal environment. These findings established a novel link between MYCN knockdown and chromatin remodeling and provided a potential therapeutic strategy for MYCN-driven neuroblastoma.

## Results

### Knockdown of MYCN Down-Regulates FACT in Neuroblastoma Cells

**Figure 1:**
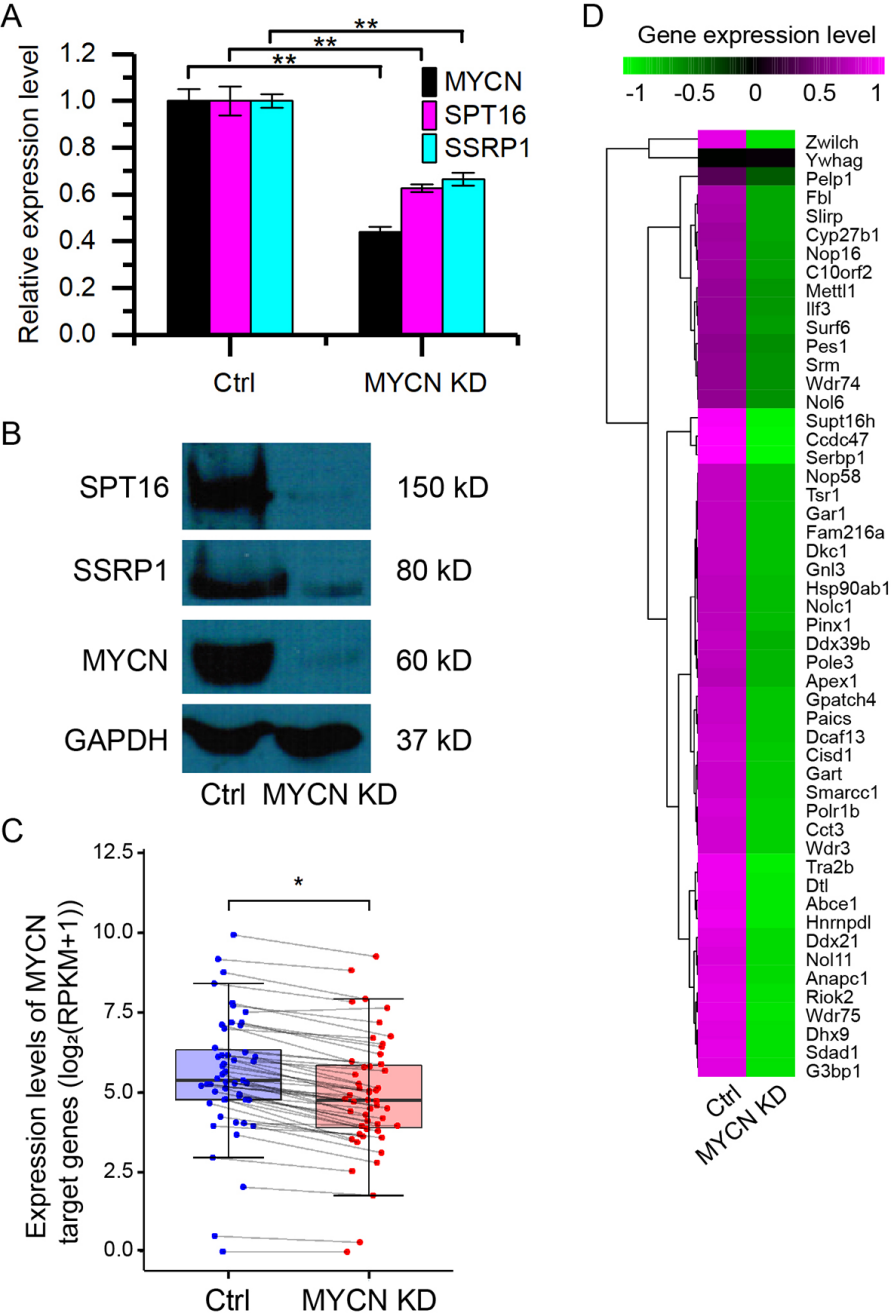
Knockdown of MYCN down-regulates the expression of FACT in neuroblastoma. **(A)** quantitative polymerase chain reaction (qPCR) results of MYCN, SPT16, and SSRP1. Error bars are SEM. (**p < 0.01, Student’s *t* test). **(B)** Western blots for MYCN, SPT16, and SSRP1 protein expression in BE(2)C cells treated with control and MYCN knockdown. **(C)** MYCN target genes are down-regulated in MYCN knockdown. (*p < 0.05, Student’s *t* test). **(D)** Heatmap showing the expression levels of the 51 MYCN target genes in BE(2)C cells treated with control and MYCN knockdown.

To investigate how MYCN regulates chromatin state, we knocked down MYCN in MYCN-amplified BE(2)C neuroblastoma cells using small interfering RNA (siRNA). The quantitative assays showed the significant decrease in the expression of MYCN and the two FACT subunits (SPT16 and SSRP1) at both mRNA and protein levels (**Figures 1A, B**). This is consistent with our previous results (Carter et al., 2015). We further profiled the genome-wide gene expressions using RNA-seq with high reproducibility (**Supplementary Figure 1A**). The results confirmed the significant decrease in the expression of *MYCN*, *SPT16*, and *SSRP1* (**Supplementary Figure 1B**). Furthermore, the previously reported 51 MYC target genes (Ji et al., 2011) in neuroblastoma tumors were significantly down-regulated as MYCN knockdown (**Figures 1C, D** and **Supplementary Figure 1C**). These results together demonstrated that MYCN knockdown was successful and consequently down-regulated FACT expression in neuroblastoma cells.

### MYCN Knockdown Disturbs Nucleosome Positioning in the Promoters and Impedes DNA Repair

**Figure 2:**
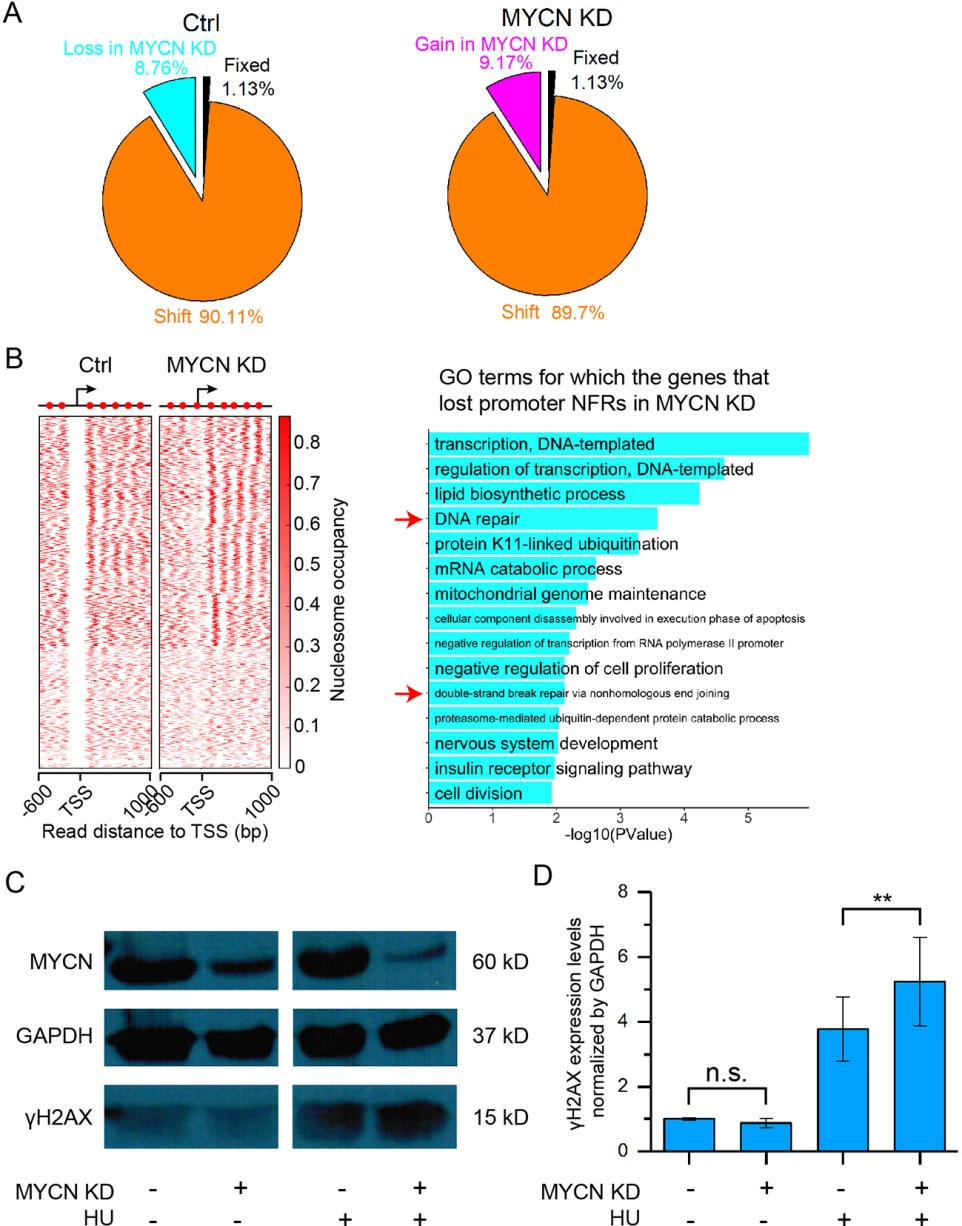
MYCN knockdown results in nucleosome remodeling and marked increase in DNA damage. **(A)** Pie charts showing the portions of fixed nucleosome, shift, loss, and gain of nucleosome. Fixed nucleosomes share the same positions before and after MYCN knockdown. Shift nucleosomes include the nucleosome pairs before and after MYCN knockdown with overlapping ≥ 30 bp and <147 bp (a nucleosome length). Nucleosome loss and gain consists of the rest of nucleosomes. **(B)** Loss of the NFRs in the promoter regions of a set of genes after MYCN knockdown through nucleosome assembly (left heatmap). Significantly enriched GO terms for this set of genes (right bar plot). **(C)** Western blots for γH2AX and MYCN protein expression in BE(2)C cells treated for 24 h with or without hydroxyurea (HU), then for another 24 h with siRNA for MYCN knockdown after hydroxyurea withdrawal. **(D)** Quantitative analysis of γH2AX and MYCN protein expression in **(C)**. (**p < 0.01, n.s., not significant, Student’s *t* test)

We next profiled genome-wide nucleosome occupancy to investigate how MYCN impacts nucleosome positioning. The nucleosome occupancy is highly reproducible (**Supplementary Figure 2A**). MYCN knockdown did not result in significantly different nucleosome occupancy on a genome scale (**Supplementary Figure 2B**). However, we observed that more than 98% of all nucleosomes that shifted were assembled or evicted in response to MYCN knockdown (**Figure 2A**). Our previous studies showed that nucleosome eviction or assembly in *cis* regulatory elements played a critical role in embryonic development, cell differentiation, etc. (Shi et al., 2014; Ye et al., 2016; Du et al., 2017; Ye et al., 2017). Therefore, we examined nucleosome positioning dynamics in the promoters when MYCN was knocked down. There exists −1, nucleosome free regions (NFRs), +1, +2, etc. canonical nucleosome arrangement around transcription start site (TSS) and gene body. The NFR locates in the upstream 200 bp to downstream 50 bp regions of TSS. If an NFR in the control sample overlapped at least 80% of a nucleosome upon MYCN knockdown, the NFR was lost in the MYCN knockdown sample, and *vice versa* (see Materials and Methods for details). The results showed that MYCN knockdown led to nucleosome assembly in a set of promoters of the genes enriched in the functions of DNA repair, double-strand break repair *via* nonhomologous end join, and nervous system development (**Figure 2B**). Consistently, MYCN knockdown resulted in a marked increase in the DNA damage marker γH2AX when the cells were treated with hydroxyurea (**Figures 2C, D**). We further obtained experiment-validated MYCN binding sites in BE(2)C cells (Zeid et al., 2018) to check whether MYCN bound promoters. The results showed that 74% of 1,099 promoters have both NFR loss upon MYCN knockdown and MYCN binding in the control sample. Taken together, this suggested that loss of the 5′ NFRs through nucleosome positioning in the promoters resulting from MYCN knockdown impeded DNA repair. In contrast, nucleosomes were evicted to form NFRs in another set of promoters that were enriched in the functions of positive regulation of cell proliferation, regulation of cytokinesis, B cell activation, and endocytosis (**Supplementary Figure 2C**).

### MYCN Knockdown Alters Chromatin State in the Promoters

In addition to nucleosome occupancy, histone modifications in the promoters are also important to gene activity. Therefore, we investigated histone modification changes in the promoters in response to MYCN knockdown. We conducted chromatin immunoprecipitation followed by parallel massive sequencing (ChIP-seq) for the key histone modifications before and after MYCN knockdown with high reproducibility (**Supplementary Figure 3A**).

**Figure 3:**
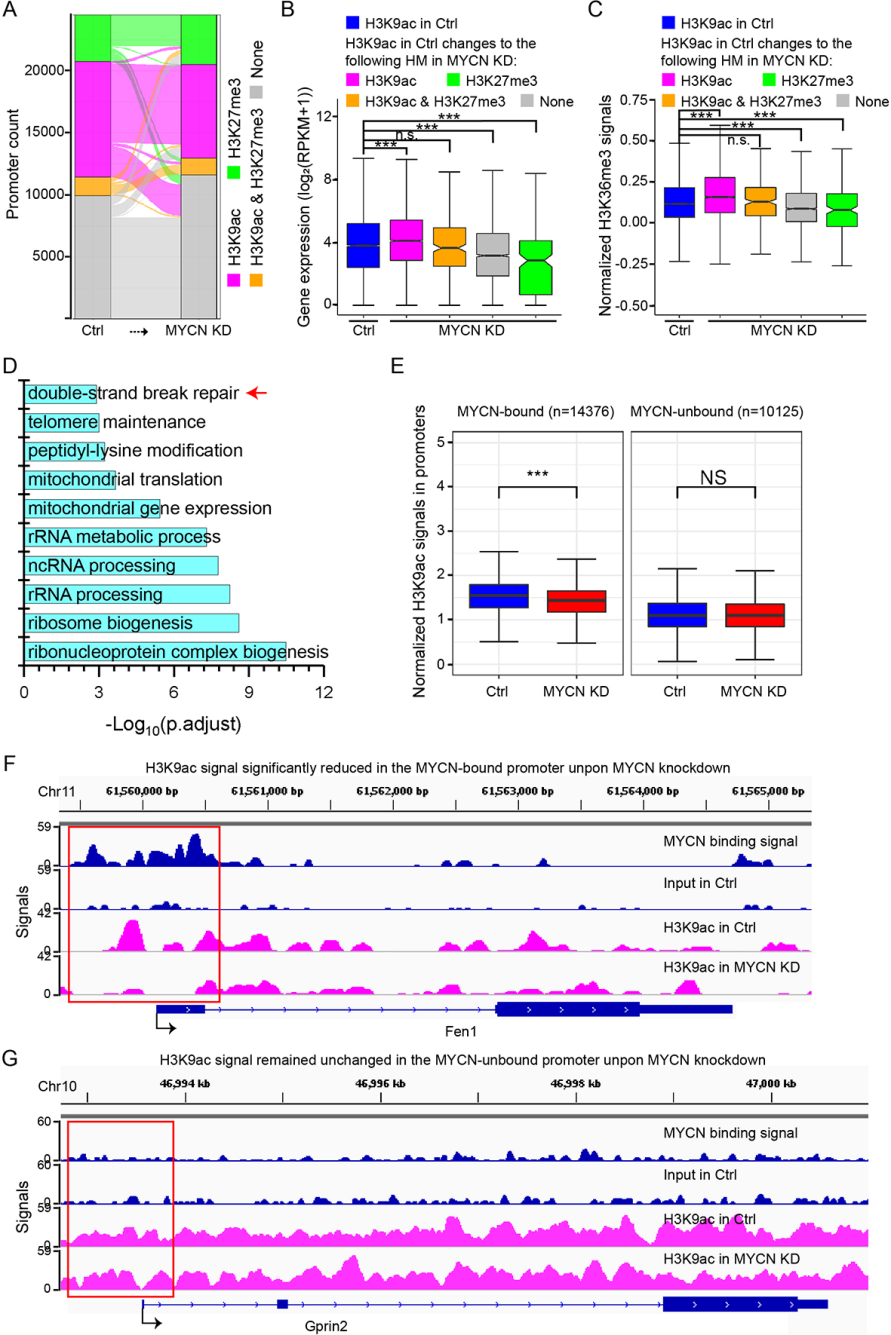
H3K9ac signals decrease in the promoters upon MYCN knockdown. **(A)** The alluvial plot shows the dynamics of histone modifications (HMs) in the promoters upon MYCN knockdown. **(B)** Changes in expression levels of the genes with H3K9ac+/H3K27me3− promoters in the control. The genes are categorized by HMs in the promoters in the MYCN knockdown sample. Overall, loss of active H3K9ac is associated with decreased expression (***p < 0.001, n.s., not significant, Wilcoxon rank sum test). **(C)** Loss of active H3K9ac is associated with decreased H3K36me3 signals in the gene body. The gene sets are the same as in **(B)** (***p < 0.001, Wilcoxon rank sum test). **(D)** Functional annotation of the genes with H3K9ac+/H3K27me3− promoters that are replaced by H3K27me3+, H3K9ac+/H3K27me3+, or none after MYCN knockdown. **(E)** H3K9ac signals are significantly reduced in the MYCN-bound promoters upon MYCN knockdown whereas H3K9ac signals remain the similar levels in the MYCN-unbound promoters (***p < 0.001, Wilcoxon rank sum test). **(F, G)** Track view of MYCN binding and H3K9ac signals in the promoter (marked in the red box) of the representative genes *Fen1* (a double-strand break repair related gene) **(F)** and *Gprin2* **(G)**.

We first explored the changes of active H3K4me3 and repressive H3K27me3 that could form bivalent domains in the promoters and played a critical role in gene transcription regulation (Kouzarides, 2007). Surprisingly, chromatin states in the promoters defined by H3K4me3 and H3K27me3 signals largely remained unchanged before and after MYCN knockdown (**Supplementary Figure 3B**). Our previous study found that another combination of histone modifications H3K9ac and H3K27me3 in the promoters were also critical to gene transcription regulation (Du et al., 2017). Therefore, we next examined the changes of H3K9ac and H3K27me3 in the promoters. H3K9ac signals were removed in many promoters (**Figure 3A**). Consequently, gene expression levels were decreased as H3K9ac signals in the promoters were removed or replaced by H3K27me3 (**Figure 3B**). H3K36me3 signals in the gene body changed in the same manner (**Figure 3C**). Interestingly, we performed gene ontology (GO) analysis of the genes with only H3K9ac in the promoters that was replaced by H3K27me3, H3K9ac/H3K27me3, or none after MYCN knockdown and identified enrichment for double-strand break repair (**Figure 3D**), suggesting that MYCN knockdown suppressed ribosome biogenesis, rRNA processing, rRNA metabolic process, and repair of DNA damage by altering chromatin state in the promoters. To confirm this, we obtained experiment-validated MYCN binding sites in BE(2)C cells (Zeid et al., 2018) and classified promoters into two groups: MYCN-bound and MYCN-unbound. Intriguingly, there was a significantly higher portion of MYCN-bound promoters whose chromatin state changed from active to non-active upon MYCN knockdown compared with the MYCN-unbound promoters (**Supplementary Figure 3C**). Further analysis found that H3K9ac signals were significantly decreased in the MYCN-bound promoters after MYCN knockdown. In contrast, H3K9ac signals remained unchanged in the MYCN-unbound promoters (**Figures 3E, G** and **Supplementary Figures 3D, E**). Of note, MYCN bound to the promoters of the genes (e.g., Fen1, Smc6, Cen1 and Rad54b) with functions of DNA damage repair. H3K9ac signals in their promoters were significantly reduced after MYCN knockdown (**Figure 3F** and **Supplementary Figure 3D**). Taken together, MYCN binding in the promoters regulated translational process or transcription of rRNA genes and the function of DNA repair by altering chromatin state.

### Histone Modification Changes in the Enhancers Upon MYCN Knockdown

Transcription-factor-bound enhancers play an important role in regulating gene expression (Bulger and Groudine, 2011). Histone modifications in enhancers control their activity. Therefore, we next investigated histone modification changes in the enhancers upon MYCN knockdown.

In order to identify enhancers and determine their chromatin state in neuroblastoma cells, we performed ChIP-seq of H3K4me1 and H3K27ac. The results were highly reproducible (**Supplementary Figure 4A**). Enhancers were predicted based on H3K4me1 signals using the tool Homer (Heinz et al., 2010). The chromatin state of enhancers was determined by the histone modifications on enhancers: active (H3K4me1+ and H3K27ac+), intermediate (H3K4me1+, H3K27ac+, and H3K27me3+), poised (H3K4me1+ and H3K27me3+), primed (H3K4me1+ only), and off (H3K4me1−).

**Figure 4:**
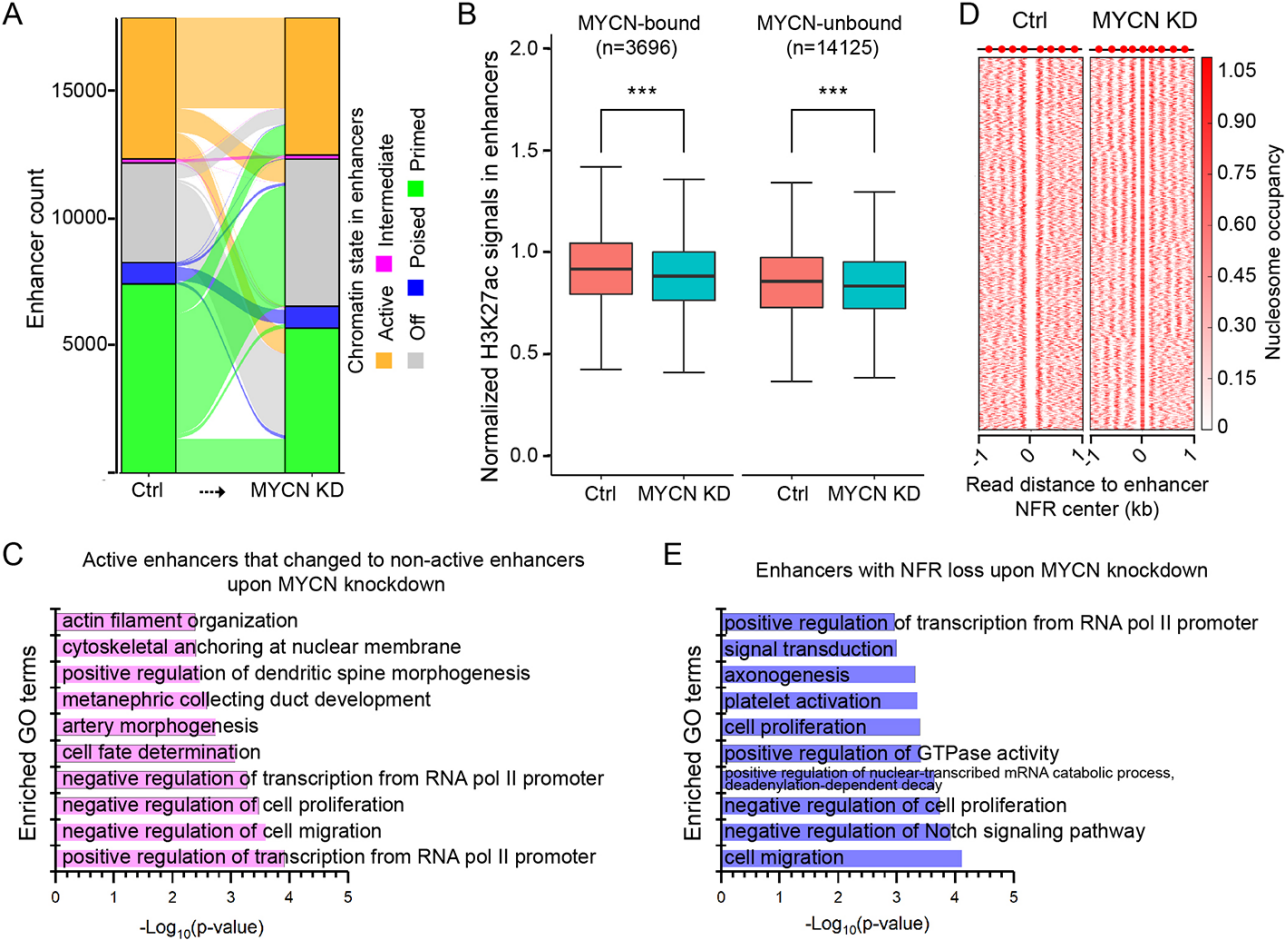
Chromatin state changes in the enhancers upon MYCN knockdown. **(A)** The alluvial plot shows the dynamics of histone modifications (HMs) in the enhancers upon MYCN knockdown. **(B)** H3K27ac signals are significantly reduced in both MYCN-bound and MYCN-unbound enhancers upon MYCN knockdown (***p < 0.001, Wilcoxon rank sum test). **(C)** GO terms for which the enhancers are enriched whose chromatin state change from active to non-active upon MYCN knockdown. **(D)** Loss of the NFRs in the enhancers after MYCN knockdown through nucleosome assembly. **(E)** GO terms for which the enhancers are enriched whose NFRs are lost upon MYCN knockdown.

Upon MYCN knockdown, most of the active, intermediate, and poised enhancers remained in the same chromatin state (**Figure 4A**). In contrast, the majority (63.6%) of primed enhancers became off enhancers, and 81.7% of off enhancers became primed enhancers (**Supplementary Figure 4B**).

To investigate the relationship between MYCN binding and chromatin state change in the enhancers upon MYCN knockdown, we used the MYCN binding sites in BE(2)C cells (Zeid et al., 2018) to categorize enhancers into MYCN-bound and MYCN-unbound two groups. Surprisingly, unlike promoters (**Figure 3E**), both H3K27ac and H3K27me3 signals were significantly changed in both MYCN-bound and MYCN-unbound enhancers upon MYCN knockdown (**Figure 4B** and **Supplementary Figure 4C**). We further did motif enrichment analysis in these two types of enhancers. As expected, the top 10 enriched motifs in MYCN-bound enhancers are all E-box (CACGTG)-related motifs such n-Myc, MNT, Max, c-Myc, NPAS, BMAL1, NPAS2, CLOCK, c-Myc, and USF1. In contrast, the top 10 enriched motifs in MYCN-unbound enhancers include Gata2, Gata6, EBF1, Lhx1, Gata1, Phox2b, Gata3, Gata4, Prop1, and Ap-2α. This indicates that transcription factors containing zinc finger domain (Gata family) and homeobox (Phox2b and Prop1) regulate MYCN-unbound enhancers.

To understand which functions were impacted through chromatin state changes in enhancers upon MYCN knockdown, we did GO analysis of the active enhancers that changed to non-active enhancers upon MYCN knockdown and identified enrichment for cell fate determination, negative regulation of cell proliferation, and negative regulation of cell migration (**Figure 4C**). Nucleosome eviction and assembly in enhancers also played a critical role in regulating gene expression (Ye et al., 2016; Ye et al., 2017). Therefore, we identified 3809 enhancers with NFR loss through nucleosome assembly upon MYCN knockdown (**Figure 4D**). The GO analysis revealed that these enhancers were enriched for cell proliferation, negative regulation of cell proliferation, and cell migration (**Figure 4E**). Additionally, the breadth of H3K4me3 domains had critical roles in regulating gene expression (Liu et al., 2016). Thus, we identified the enhancers whose width became broader and that remained active upon MYCN knockdown. The GO analysis revealed that these enhancers were enriched for cell division, cell migration, and cell–cell adhesion (**Supplementary Figure 4D**). Notably, all these GO terms were related to cancer initiation and progression. Collectively, MYCN knockdown altered chromatin state in the enhancers in multiple manners and impacted functions related to tumorigenesis and cancer progression.

### MYCN Knockdown Independently Alters Chromatin State in the Promoters and the Enhancers

Genome-wide Hi-C study showed that chromatin loops mediated enhancer–promoter interactions. Our previous study revealed that chromatin state changes in the enhancers and the promoters regulated gene expression in synergy (Ye et al., 2017). Thus, we further investigated whether there was a correlation between chromatin state changes in these two type of *cis* regulatory elements upon MYCN knockdown. We first associated the nearest promoter within 100 kb to each enhancer. Next, we grouped the enhancers by chromatin change upon MYCN knockdown. Then, we classified the associated promoters by chromatin state change and counted the promoters for each group of enhancers. The results showed no correlation between chromatin state changes in the promoters and the enhancers (**Table 1**). Similarly, we grouped the promoters by chromatin change upon MYCN knockdown and examined chromatin change in the associated enhancers for each group of promoters. The results also showed no correlation between chromatin state changes in the promoters and the enhancers (**Table 2**). Collectively, these findings suggested that chromatin states in the promoters and the enhancers independently changed upon MYCN knockdown.

**Table 1:** Statistics of categorized promoters associated with enhancers classified by chromatin state change upon MYCN knockdown.

Enhancer chrom state change upon MYCN KD	Count of the associated promoters categorized by chrom state change		
	Active → non-active	Non-active → active	*p* value
Off → primed	361	169	0.74
Primed → off	494	241	
Non-active → active	143	80	0.68
Active → non-active	193	100	
Narrow → wide	45	26	0.62
Wide → narrow	85	57	

p value, chi-square test.

**Table 2:** Statistics of categorized enhancers associated with promoters classified by chromatin state change upon MYCN knockdown.

Promoter chrom state change upon MYCN KD	Count of the associated enhancers categorized by chrom state change		
	Active → non-active	Non-active → active	*p* value
Non-active → active	246	167	0.14
Active → non-active	421	343	
Promoter chrom state change upon MYCN KD	Count of the associated enhancers categorized by chrom state change		
	Primed → off	Off → primed	*p* value
Non-active → active	671	421	0.95
Active → non-active	1387	866	
Promoter chrom state change upon MYCN KD	Count of the associated enhancers categorized by chrom state change		
	Wide → narrow	Narrow → wide	*p* value
Non-active → active	120	49	0.99
Active → non-active	183	75	

p value, chi-square test.

## Discussion

Chromatin structure controls the binding of myriad transcription factors. Aberrant chromatin structures result in a state of “epigenetic instability”. Accordingly, the gene expressions are altered and the differentiation and proliferation programs are perturbed. As a result, these aberrant changes of chromatin structures predispose to oncogenic transformation. In this study, the epigenomics analyses revealed that MYCN knockdown changed nucleosome organization and chromatin states in the promoters and the enhancers likely through regulating target genes. As a result, repair of DNA damage was impaired. These findings revealed MYCN’s distinct function of chromatin remodeling other than its roles as a classical transcription factor. Since *MYCN* is an important oncogene and its amplification is observed in many cancers, the role of chromatin remodeling for MYCN may be conserved in other cancers. This study provided the molecular basis for potential neuroblastoma therapy approach through chromatin remodeling.

MYCN, as a classical transcription factor, played a pivotal role in tumorigenesis and cancer progression that has been extensively studied. MYCN and its partner Max formed a heterodimer and bound to the conserved regulatory element E-box of the target genes to regulate their expression (Gherardi et al., 2013). As a result, MYCN regulated a variety of cancer-related biological processes such as apoptosis, angiogenesis, invasion, and metabolism (Bell et al., 2010; Westermark et al., 2011). For example, MYCN regulated neuroblastoma initiation by activating polycomb protein BMI1 (Ochiai et al., 2010) that decreased p53 protein stability (Calao et al., 2013). Although MYCN regulated some target genes through recruiting or interacting with chromatin remodeling complex (Cotterman et al., 2008; Gherardi et al., 2013), it remained elusive whether MYCN had a function of chromatin remodeling. This study demonstrated that MYCN played a role in regulating nucleosome positioning and histone modifications in both the proximal and the distal regulatory regions in neuroblastoma cells BE(2)C. Therefore, these findings implicated a novel epigenetic regulatory role of MYCN regardless of its transcription regulatory role as a classical transcription factor.

MYCN regulated many target genes including histone chaperones. For example, our previous study found that FACT was an MYCN target gene. Moreover, MYCN and FACT expression formed a positive feedback loop in neuroblastoma cells. FACT inhibition caused cell death *in vitro* by facilitating cancer cell death through blocking repair of DNA damage (Carter et al., 2015). Consistently, our results showed that MYCN knockdown down-regulated expression of the two subunits of FACT and resulted in marked increase in DNA damage in neuroblastoma cells. However, we still lack evidence whether MYCN alters chromatin structures mainly through its forward feedback loop with FACT. Moreover, it is not fully understood how this chromatin remodeling affects the specific DNA repair pathways. Further in-depth studies will be required to address these questions.

## Materials and Methods

### Cell Culture and RNAi

The human neuroblastoma cell line BE(2)C was cultured in Dulbecco’s Modified Eagle’s Medium (DMEM) (Life Technologies 11995065) supplemented with 10% FBS (fetal bovine serum) at 37°C with 5% CO_2_. siRNAs used were non-targeting control siRNA (Qiagen 1027281) and MYCN (Qiagen SI03087518).

On the first day, cells were cultured in 60-mm dishes, ∼4 × 10^5^ cells/dish. On the second day, 4 μl of 20 mM siRNA and 16 μl of Lipofectamine 2000 (Life Technologies 11668019) were incubated with 1 ml of Optimem (Life Technologies 51985034) at RT (room temperature) for 5 min, respectively. Then, these two 1-ml volumes of Optimem were mixed and left at RT for 20 min. DMEM was removed from the cells cultured on the first day. The cells were washed once with PBS (Life Technologies 10010023). Add the above 2 ml of Optimem mixed with siRNA and Lipofectamine 2000 to the dish at 37°C for 6 h. Add 2 ml of DMEM supplemented with 20% FBS to the dish. At this point, there was a total of 4 ml of mixture, and the final siRNA concentration was 40 nM. On the third day, the cells were collected for later use.

### mRNA Extraction and qRT-PCR

The total RNA was isolated using RNAsimple Total RNA Kit (TIANGEN DP419), and the RNA concentration was measured using NanaDrop 2000 (Thermo Scientific). The integrity of RNA was tested by running AGE (agarose gel electrophoresis). Reverse transcription of RNA utilized PrimeScript^™^ RT Master Mix (TAKARA RR036A), subsequently followed by real-time PCR using SYBR^®^Premix Ex Taq^™^ (TAKARA RR420A). Relative abundance of mRNA transcript for the selected genes was calculated using the ΔΔCt approximation relative to housekeeping gene GAPDH. The real-time PCR primers were MYCN-F 5′-ACAGTGAGCGTCGCAGAAAC-3′, MYCN-R 5′-AGCAAGTCCGAGCGTGTTC-3′, GAPDH-F 5′-AATCCCATCACCATCTTCC-3′, and GAPDH-R 5′-CATCACGCCACAGTTTCC-3′.

### Antibodies

The antibodies used for ChIP were IgG (cell signaling 2729), H3K4me1 (Abcam ab8895), H3K4me3 (Abcam ab8580), H3K9ac (Abcam ab10812), H3K27ac (Abcam ab4729), H3K27me3 (Active motif 39155), and H3K36me3 (Abcam ab9050). The following antibodies were used for Western blot: mouse anti-SPT16 (BioLegend 607002, 1:1000), mouse anti-SSRP1 (BioLegend 609702, 1:500), mouse anti-MYCN (Santa Cruz Biotechnology Sc53993, 1:1000), mouse anti-γH2AX (Abcam ab26350, 1:1000), mouse anti-GAPDH (Abcam ab8245, 1:10000), and anti-mouse HRP (Thermo Scientific 32430, 1:1500).

### Western Blotting

The total protein was isolated in RIPA lysis buffer, and protein concentration was measured by using Enhanced BCA Protein Assay Kit (Beyotime P0010S). Equivalent amounts of protein were separated by SDS-PAGE and blocked by standard methodologies. Immobilon-NC Transfer Membrane (Millipore HATF00010) was successively incubated with primary antibody at 4°C overnight and with second antibody at RT for 1 h. Enhanced chemiluminescence agent was SuperSignal West Pico Chemiluminescent Substrate (Thermo Scientific 34087), which was used according to the manufacturer’s instructions. GAPDH was used as the internal reference. Films were analyzed using Image J (NIH), and all samples were normalized to the control sample and reference gene GAPDH.

### DNA Damage and Repair

On the first day, cells were cultured in 60-mm dishes, ∼4 × 10^5^ cells/dish. Hydroxyurea (HU) (Selleck S1896) was added to DMEM in the dish, and the final concentration was 2 mM. On the second day, DMEM containing HU was removed and 2 ml of Optimem mixed with siRNA and Lipofectamine 2000 was added to the dish. Other operations were the same with RNAi.

### MNase-Seq and Chip-Seq

A total of ∼1 × 10^7^ cells were cross-linked with 1% formaldehyde at RT for 8 min, and the cross-linking was terminated by adding 0.125 M glycine at RT for 5 min. Cells were collected for lysis to isolate nuclei. Suspend the nuclei in 1 ml of MNase digestion buffer [10 mM Tris–HCl, pH 7.5, 15 mM NaCl, 60 mM KCl, 1 mM CaCl_2_, 0.15 mM spermine, 0.5 mM spermidine, and 1× PI (Roche 04693132001)]. Add 3 μl of MNase (Micrococcal Nuclease; NEB M0247S) at 37°C for 25 min. The digestion was terminated by adding EDTA to a final concentration of 10 mM. Reverse cross-linking by adding proteinase K at 65°C for 2–4 h. Mononucleosomal DNA fragments were purified using phenol-chloroform and examined by running AGE.

Approximately 15 μg of nucleosomal DNA and 3–5 μg of histone modification (HM) antibodies were mixed together with 430 μl of ChIP buffer (20 mM Tris–HCl, pH 8.1, 2 mM EDTA, 150 mM NaCl, 0.1% Triton X-100, and 1× PI) for chromatin immunoprecipitation. The mixture was incubated overnight at 4°C with rotation. Twenty-five microliters of ChIP-Grade Protein G Magnetic Beads (Cell Signaling 9006S) was added to the mixture for another 2 h rotation at 4°C. The beads were successively washed three times by low salt wash buffer (20 mM Tris–HCl, pH 8.1, 2 mM EDTA, 150 mM NaCl, 1% Triton X-100, and 0.1% SDS), and once by high salt wash buffer (20 mM Tris–HCl, pH 8.1, 2 mM EDTA, 500 mM NaCl, 1% Triton X-100, and 0.1% SDS). ChIP’ed DNA fragments were eluted by ChIP elution buffer (50 mM Tris–HCl, pH 8.0, 10 mM EDTA, and 0.3% SDS) at 65°C for 50 min and purified using phenol-chloroform.

The purified mononucleosomal DNA fragments by MNase digestion and the ChIP’ed DNA fragments on the nucleosomes with HMs by HM antibodies were subjected to massively parallel DNA sequencing on Illumina HiSeq2000 platform using 125-bp pair-end protocol, respectively.

### RNA-Seq Data Analysis

RNA-seq reads were mapped to hg19 genome assembly and transcriptome by Hisat2 (Kim et al., 2015). Read counts of RefSeq annotated genes were calculated using featureCounts (Liao et al., 2014). The read count matrix was input into DESeq2 (Love et al., 2014) to model the reads distribution and then the unwanted variations of the data were removed by RUVSeq package (Risso et al., 2014). The expression values of known RefSeq genes were calculated as Reads Per Kilobase per Million mapped reads (RPKM) using the normalized read counts of RUVSeq (Risso et al., 2014).

Gene expression signal tracks for the control and MYCN knockdown samples were generated from RNA-seq alignments of combined biological replicates using Deeptools2 (Ramirez et al., 2016) in 10-bp bins. The signals were normalized as RPKM.

### Chip-Seq and MNase-Seq Data Analysis

Raw reads of ChIP-seq and MNase-seq data were trimmed for adapter sequences and low-quality bases using Trim Galore software. Then, the clean reads were mapped to hg19 genome assembly by Bowtie2 (Langmead and Salzberg, 2012), and only the proper pairs with high mapping quality (mapq > 10) were retained for further analysis. PCR duplicates were removed with Samtools (Li et al., 2009).

Genome-wide comparisons of biological replicates were processed as follows: First, the genome was divided into non-overlapping 10-kb bins. Second, the read pairs’ midpoints were assigned to the bins for each biological replicate. Third, read count of each bin was calculated and normalized as RPKM. Then, the Pearson correlation coefficients between biological replicate pairs were calculated to measure the reproducibility of biological replicates.

As the reproducibility of biological replicates was high in ChIP-seq and MNase-seq, we pooled the biological replicates of each sample in further analysis.

We generated signal tracks of ChIP-seq and MNase-seq data using Deeptools2 (Ramirez et al., 2016) in 10-bp bins. The signals were normalized as RPKM.

### Nucleosome Prediction and Positioning Dynamics Upon MYCN Knockdown

The midpoint position of proper read pairs of MNase-seq data was used as nucleosome dyad (termed as index) coordinate. Then, we used Genetrack (Albert et al., 2008) software to predict the nucleosome positions and calculate occupancy (the normalized read count) and fuzziness of each nucleosome from the index coordinate distributions. The fuzziness value was defined as the standard deviation of index coordinate’s distances to the predicted nucleosome dyad. The predicted nucleosomes with low read count (rc < 6) were filtered out to reduce false discovery rates.

The nucleosome positioning dynamics upon MYCN knockdown was analyzed as previous studies (Ye et al., 2016; Ye et al., 2017) with minor modifications. The two closest nucleosomes in the control and MYCN knockdown samples were retained nucleosome pairs. For each nucleosome pair, the nucleosomes whose locations were not changed were defined as the fixed nucleosomes. The nucleosomes were shifted if their midpoint distance was ≥1 bp and <117 bp; that is, the two nucleosomes overlapped ≥30 bp and <147 bp (a nucleosome length). The rest of the nucleosomes were gained or lost upon MYCN knockdown.

According to the composite distribution of nucleosomes relative to the TSS, the upstream 200-bp to downstream 50-bp regions of TSSs were the canonical nucleosome-free regions (NFRs). If an NFR in the control sample overlapped at least 80% of a nucleosome upon MYCN knockdown, the NFR was lost in the MYCN knockdown sample. Opposite to this, the NFR formed upon MYCN knockdown. We plotted nucleosome occupancy in the regions around these two sets of NFRs as heat maps and clustered it by *K*-means (*K* = 5).

### Chromatin States of Genome Regions Determined by Histone Modifications

Chromatin states were identified and characterized by ChromHMM v1.15 (Ernst and Kellis, 2012) with distinct histone modification combinations. The genome was divided into 200-bp bins. We used BinarizedBam command to calculate H3K9ac, H3K4me3, H3K27me3, and H3K27ac signals with IgG samples as input for each bin, respectively. Then, the chromatin states were trained and learned from these bins with four emission states (two individual histone modifications, one combined histone modifications, none) in each of these three histone modification combinations (H3K9ac with H327me3, H3K4me3 with H3K27me3, and H3K27ac with H3K27me3). Finally, we got segments with four chromatin states genome-wide for each histone modification combination.

### Chromatin State Changes in the Promoters Upon MYCN Knockdown

Promoters were defined as the upstream 2-kb regions to the downstream 1-kb regions of TSS. We took genomic segments classified by ChromHMM to define the chromatin states of promoters. The promoters that contain an H3K9ac+/H3K27me3+ segment, or both H3K9ac+ only segments and H3K27me3+ only segments were defined as H3K9ac+/H3K27me3+ promoters. The promoters that exclusively contain an H3K9ac+ only segments or H3K27me3+ only segments were defined as H3K9ac+ or H3K27me3+ promoters, respectively. The rest of the promoters were defined as no marked promoters. The chromatin states in the promoters for the combination of H3K4me3 with H3K27me3 were classified in the same way.

### Enhancer Predictions and Characterization

H3K4me1 peaks were identified using findPeaks command from Homer (Heinz et al., 2010) with the following parameter setting: -style histone -F 2. Peaks located within 2-kb regions of known TSSs were excluded to avoid overlap with the promoters. For two peaks from the two samples that overlapped at least 50% of the shorter peak, the peak with higher Homer score was retained. The non-overlapping peaks were all retained as well. Peaks in ctrl and MYCN KD were merged using the following strategy: Overlap peaks (at least 50% overlap) with higher peak score were left and pooled with non-overlap peaks in each sample. The final peak set retained in this manner was treated as enhancers for further analysis. Enhancers with H3K4me1 peak(s) were at “On” state. In contrast, enhancers without a H3K4me1 peak were at “Off” state.

For the enhancers defined by the overlapping H3K4me1 peaks from the two samples, we examined the length of the original peaks in each sample: If the peak length in the MYCN knockdown sample was over 1.5-fold wider than that in the control sample, this enhancer became broader upon MYCN knockdown. Similarly, if the peak length in the MYCN knockdown sample was over 1.5-fold shorter than that in the control sample, this enhancer became shorter upon MYCN knockdown.

We took the ChromHMM segments classified by the combination of H3K27ac with H3K27me3 as above to define the chromatin states of the “On” enhancers. The enhancers that contain an H3K27ac+/H3K27me3+ segment, or both H3K27ac+ only segments and H3K27me3+ only segments had an intermediate state. The enhancers that exclusively contain an H3K27ac+ only segment had an active state. The enhancers that exclusively contain an H3K27me3+ only segment had a poised state. The rest of “On” enhancers had a primed state.

The nearest TSS within 50-kb regions of an enhancer was defined as the enhancer target gene. Thus, a gene may associate with several enhancers.

The ggalluvial package in R was used to show the chromatin state dynamics in the promoters and the enhancers with alluvial plots.

### Analysis of MYCN Chip-Seq Data in BE(2)C Cells

We downloaded the public MYCN ChIP-seq data in BE(2)C cells (Zeid et al., 2018) and mapped the reads to hg19 genome assembly. MYCN peaks were called using MACS2 v2.1.1.20160309 (Liu, 2014) with default parameters. The promoters and the enhancers were classified as “MYCN-bound” if they contain at least one MYCN peak. The rest of the promoters and the enhancer were classified as “no MYCN-bound”.

### Functional Annotations

The GO term analysis of the genes with loss or gain in NFR in the promoter regions after MYCN knockdown (**Figures 2B** and **S2C**), of the genes whose enhancers changed from active to non-active, of the genes with NFR loss in the enhancers upon MYCN knockdown, and of the genes whose enhancers became broader and remained active upon MYCN knockdown (**Figures 4C, D** and **Supplementary Figure 4D**) was carried out using the tool DAVID v6.8 (Huang et al., 2009) (https://david.ncifcrf.org). The GO term analysis of the genes with H3K9ac+/H3K27me3− promoters that are replaced by H3K27me3+, H3K9ac+/H3K27me3+, or none after MYCN knockdown (**Figure 3C**) was performed with clusterProfiler (Yu et al., 2012).

## Data Availability

The RNA-seq, MNase-seq, and ChIP-seq data sets have been deposited in the Gene Expression Omnibus under the accession number GSE120859.

## Author Contributions

WZ did most of the data analyses and XH did most of the experiments. QZ, ZH, and XZ helped with bioinformatics analyses. LG and YD helped with the experiments. DC, BC, and AQ helped with the study initiation. CJ conceived the study. WZ, XH, and CJ wrote the manuscript. All authors read and approved the final manuscript.

## Funding

This work was supported by the National Key Research and Development Program of China (Grant No. 2016YFA0100400), National Natural Science Foundation of China (Grant Nos. 31771419, 31721003 and 31800858), China Postdoctoral Science Foundation (Grant No. 2017M621526).

## Conflict of Interest Statement

The authors declare that the research was conducted in the absence of any commercial or financial relationships that could be construed as a potential conflict of interest.
