# Supplementary material for: Increase in DNA Damage by MYCN Knockdown Through Regulating Nucleosome Organization and Chromatin State in Neuroblastoma
Source: Front Genet. 2019 Jul 24;10:684. doi: 10.3389/fgene.2019.00684 (PMC6667652; doi:10.3389/fgene.2019.00684)
Supplement: Supplementary file 1 [file DataSheet_1.pdf]

Supplementary Figures and Figure legend

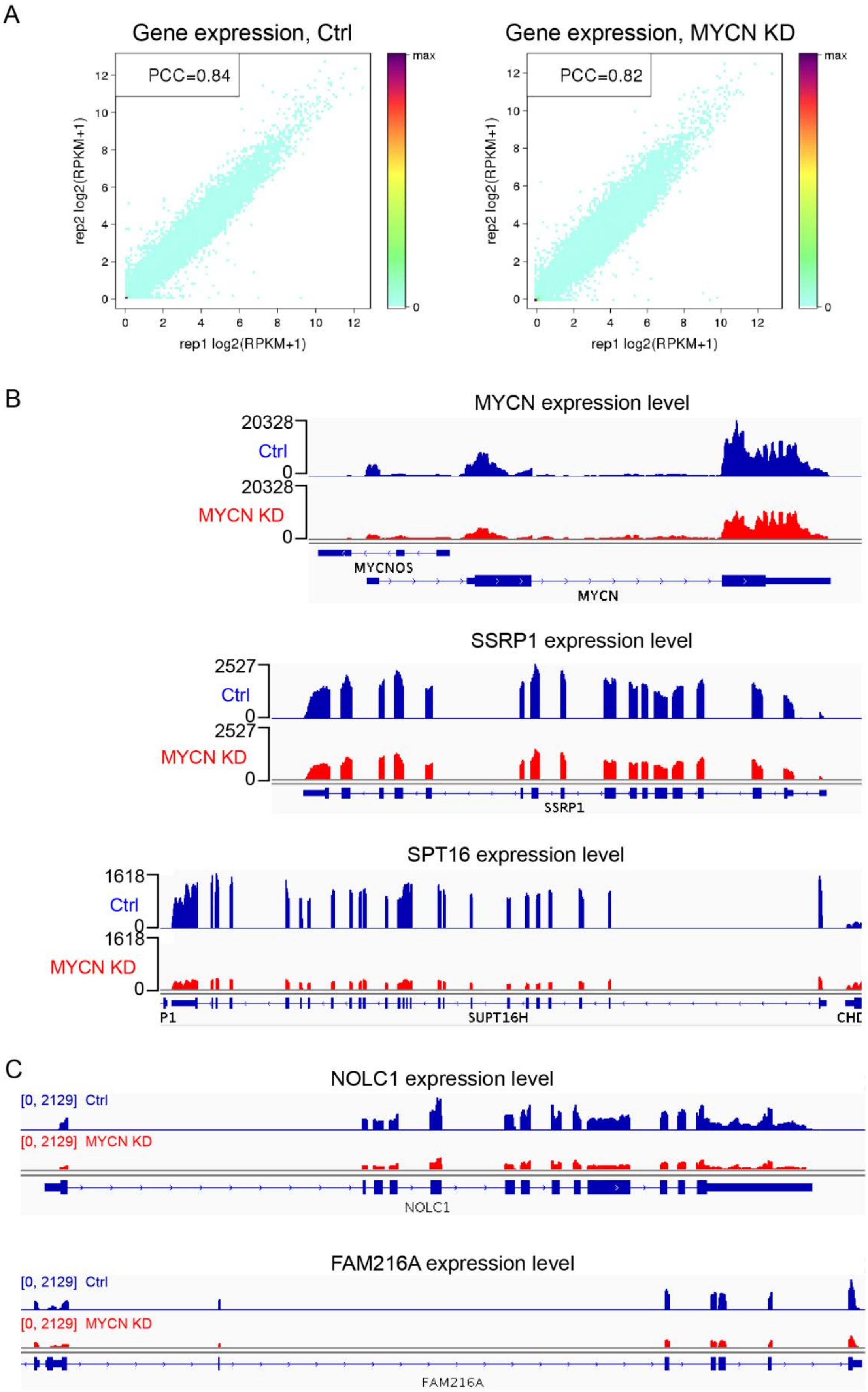

Supplementary Figure S1 | Knockdown of MYCN down-regulates the expression of FACT in neuroblastoma. **(A)** RNA-seq data are highly reproducible. **(B)** Track view of RNA-seq density shows greatly decreased expression levels of MYCN, SPT16, and SSRP1 after MYCN knockdown. **(C)** Track view of RNA-seq density shows greatly decreased expression levels of MYCN target genes *Nolc1* and *Fam216a* after MYCN knockdown.

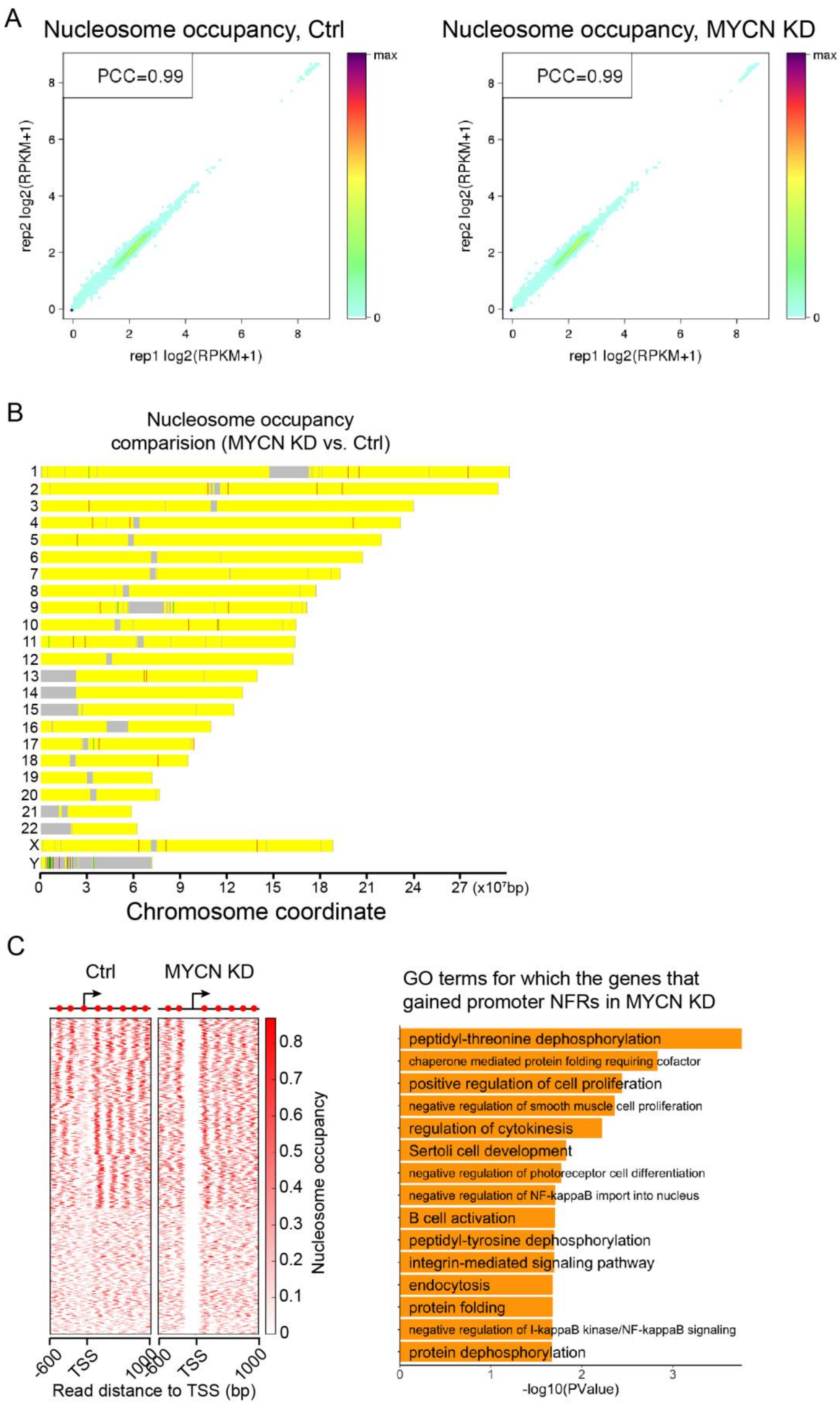
A
Nucleosome occupancy, Ctrl
PCC=0.99
rep2 log2(RPKM+1)
rep1 log2(RPKM+1)
max
0
Nucleosome occupancy, MYCN KD
PCC=0.99
rep2 log2(RPKM+1)
rep1 log2(RPKM+1)
max
0
B
Nucleosome occupancy comparision (MYCN KD vs. Ctrl)
1
2
3
4
5
6
7
8
9
10
11
12
13
14
15
16
17
18
19
20
21
22
X
Y
0
3
6
9
12
15
18
21
24
27 (x10⁷bp)
Chromosome coordinate
C
Ctrl
MYCN KD
Nucleosome occupancy
Read distance to TSS (bp)
-600
TSS
1000
GO terms for which the genes that gained promoter NFRs in MYCN KD
peptidyl-threonine dephosphorylation
chaperone mediated protein folding requiring cofactor
positive regulation of cell proliferation
negative regulation of smooth muscle cell proliferation
regulation of cytokinesis
Sertoli cell development
negative regulation of photoreceptor cell differentiation
negative regulation of NF-kappaB import into nucleus
B cell activation
peptidyl-tyrosine dephosphorylation
integrin-mediated signaling pathway
endocytosis
protein folding
negative regulation of I-kappaB kinase/NF-kappaB signaling
protein dephosphorylation
-log10(PValue)

Supplementary Figure S2 | Global nucleosome occupancy profiles. **(A)** The genome is scanned with a 10-kb window and nucleosome occupancy is calculated and normalized as RPM (Reads Per Million mapped reads) for each window. The high correlation of the global nucleosome occupancy between the biological replicates indicates the high reproducibility (PCC: Pearson Correlation Coefficient). **(B)** Limited variations in nucleosome occupancy across the genome after MYCN knockdown compared with the control. The ratio of normalized nucleosome occupancy within each window is presented by a color. Red indicates read count in MYCN knockdown is at least 2 fold of that in the control. Green indicates read count in MYCN knockdown is less than half of that in the control. Yellow indicates the ratio is less than two folds. Grey indicates regions of Ns in the genome. **(C)** NFR formation in the promoter regions of a set of genes after MYCN knockdown through nucleosome eviction (left heatmap). Significantly enriched GO terms for this set of genes (right bar plot).

A

Pearson correlation coeffcients of biological replicates

	Ctrl	MYCN KD
H3K4me3	0.9976	0.9963
H3K27me3	0.9981	0.9964
H3K9ac	0.9982	0.9975
H3K36me3	0.9977	0.9962
IgG	0.9973	0.996

B

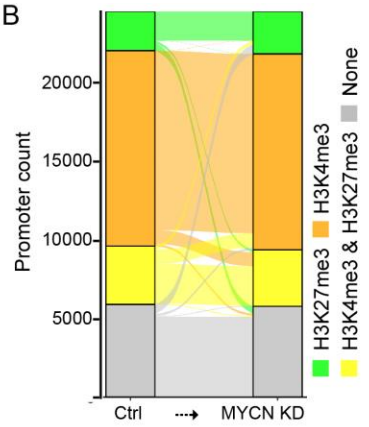

C

The number of the categorized promoters

	MYCN-bound	MYCN-unbound
active → non-active	2583	385
other	11793	9740

D

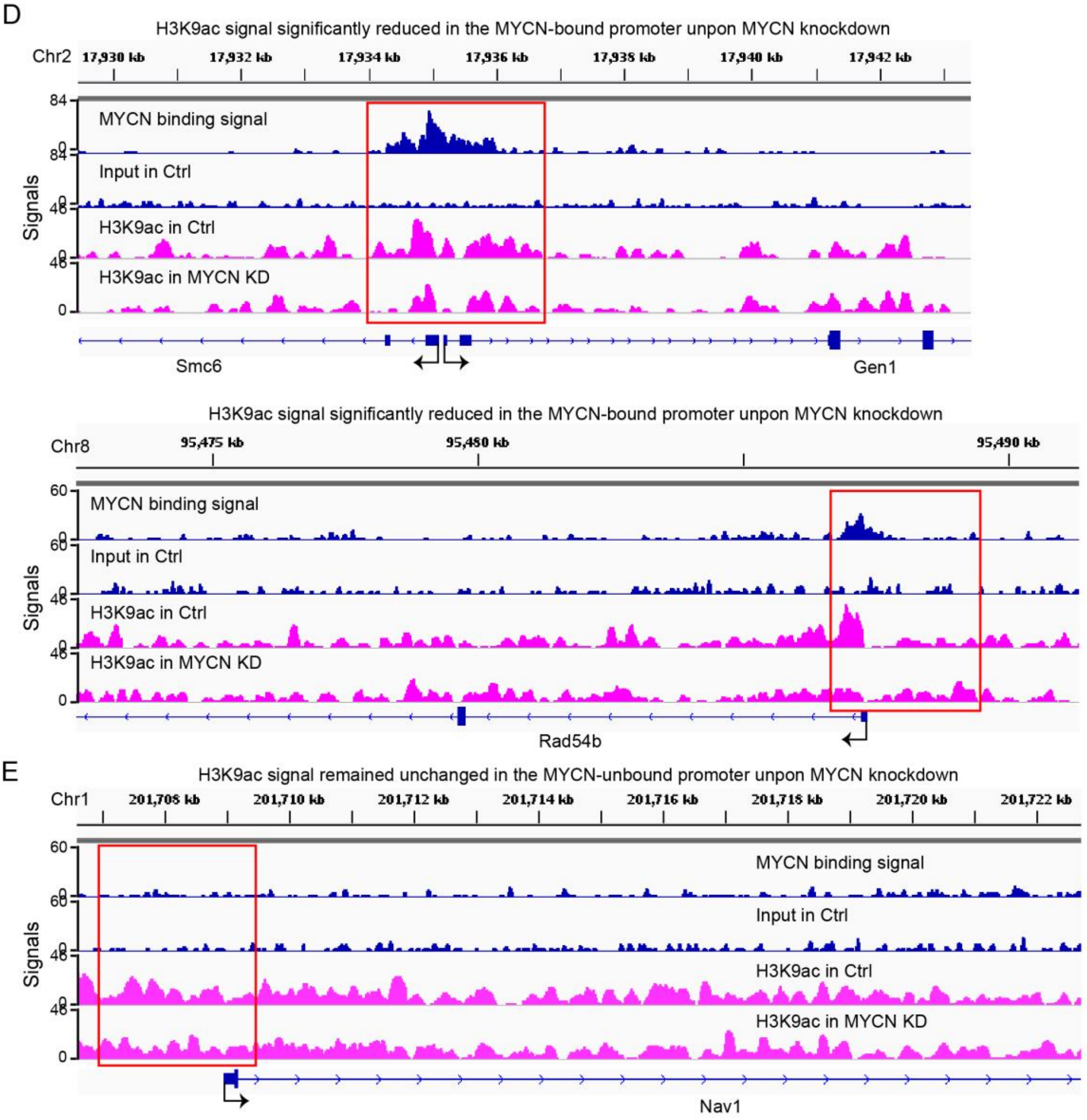

Supplementary Figure S3 | Histone modification changes in the promoters upon MYCN knockdown. **(A)** The ChIP-seq data of histone modifications are highly

reproducible. **(B)** The alluvial plot shows the dynamics of histone modifications (HMs) in the promoters upon MYCN knockdown. **(C)** There are significantly higher portion of MYCN-bound promoters than MYCN-unbound promoters whose chromatin state changes from active to non-active upon MYCN knockdown ($p < 0.001$, Chi square test). **(D)** Track view of MYCN binding and H3K9ac signals in the MYCN-bound promoter (marked in the red box) of the representative DNA repair-related genes *Smc6*, *Cen1*, *Rad54b*. **(E)** Track view of MYCN binding and H3K9ac signals in the MYCN-unbound promoter (marked in the red box) of the representative gene *Nav1*.

A

Pearson correlation coeffcients of biological replicates

	Ctrl	MYCN KD
H3K27ac	0.9983	0.9974
H3K4me1	0.9984	0.9978
IgG	0.9973	0.996

B

The statistics of the enhancers categorized by the chromatin state changes

Enhancer chrom state in Ctrl	Enhancer count in MYCN KD					
	active (%)	intermediate (%)	poised (%)	primed (%)	off (%)	Total (%)
active	3542 (64.1)	4 (0.1)	32 (0.6)	1015 (18.4)	930 (16.8)	5523 (100)
intermediate	6 (3.7)	118 (72.8)	32 (19.8)	3 (1.9)	3 (1.9)	162 (100)
poised	24 (2.9)	27 (3.2)	537 (64.3)	131 (15.7)	116 (13.9)	835 (100)
primed	1180 (15.9)	5 (0.1)	164 (2.1)	1346 (18.2)	4710 (63.6)	7405 (100)
off	622 (16.0)	1 (0.03)	92 (2.4)	3181 (81.7)	0 (0.0)	3896 (100)
Total	5374 (30.2)	155 (0.9)	857 (4.8)	5676 (31.9)	5759 (32.3)	17821 (100)

C

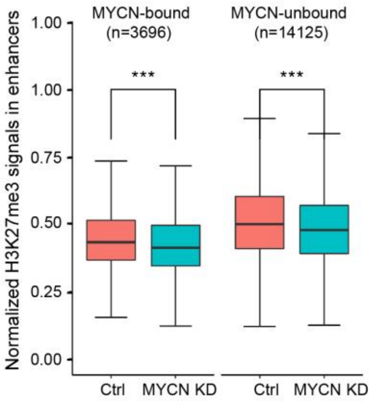

D

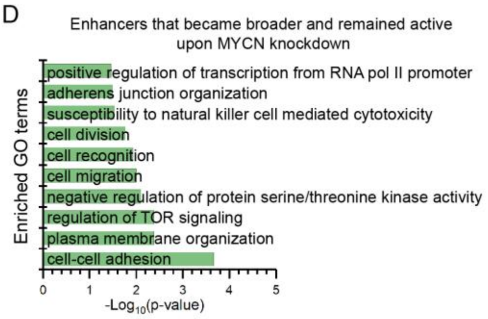

Supplementary Figure S4 | Histone modification changes in the enhancers upon MYCN knockdown. **(A)** The ChIP-seq data of histone modifications are highly reproducible. **(B)** The statistics of the enhancers categorized by the chromatin state changes upon MYCN knockdown. **(C)** H3K27me3 signals are significantly reduced in both MYCN-bound and MYCN-unbound enhancers upon MYCN knockdown (***: $p < 0.001$, Wilcoxon rank sum test). **(D)** GO terms for which the enhancers are enriched whose width becomes larger and remains an active chromatin state upon MYCN knockdown.
